# Meta-Analysis of Xihuang Pill Efficacy When Combined with Chemotherapy for Treatment of Breast Cancer

**DOI:** 10.1155/2019/3502460

**Published:** 2019-03-12

**Authors:** Dan Mao, Lei Feng, Siqi Huang, Shaofan Zhang, Weijun Peng, Sifang Zhang

**Affiliations:** ^1^Department of Integrated Traditional Chinese and Western Internal Medicine, The Second Xiangya Hospital of Central South University, Changsha, Hunan 410000, China; ^2^Department of Oncology, Hunan Academy of Traditional Chinese Medicine Affiliated Hospital, Changsha, Hunan 410006, China; ^3^Department of Oncology, Yueyang Hospital of Traditional Chinese Medicine, Yueyang, Hunan 414000, China

## Abstract

**Objective:**

To systematically evaluate the efficacy of Xihuang pill (XHP) in breast cancer patients receiving chemotherapy.

**Methods:**

Three English and four Chinese databases were searched. Literature was screened using EndNote X7 and data were analyzed by Review Manager.

**Results:**

This review included 13 randomized clinical studies of 1272 patients. The results showed that XHP increased the tumor response [risk ratio (RR) = 2.91; 95% confidence interval (CI): 1.98-4.26] and improved Karnofsky performance score (KPS) for breast cancer patients receiving chemotherapy [RR = 4.96; 95% CI = 2.07-11.86]. In addition, XHP treatment significantly reduced chemotherapy-induced adverse events, including nausea and vomiting [RR = 0.50; 95% CI = 0.33-0.74], WBC reduction [RR = 0.71; 95% CI = 0.47-1.06], platelet reduction [RR = 0.53; 95% CI = 0.19-1.44], hemoglobin reduction [RR = 0.31; 95% CI = 0.19-0.52], and hepatic function damage [RR = 0.63; 95% CI = 0.35-1.11].

**Conclusion:**

XHP combined with chemotherapy in comparison with chemotherapy alone could significantly enhance the tumor response, improve KPS, and alleviate toxicity induced by chemotherapy in breast cancer patients.

## 1. Introduction

Breast cancer is one of the most common types of malignant tumors among women worldwide and is also the leading cause of cancer death among women in the world [1]. Approximately 252,710 new cases of invasive breast cancer and 40,610 breast cancer deaths are expected to occur among US women in 2017 [2]. In the developing world, the incidence rate of breast cancer has been increasing due to extended life expectancies, developing urbanization, and the adoption of stressful modern lifestyles [3]. Surgery [4], chemotherapy [5], endocrinotherapy [6], molecular targeted therapy [7], and immunotherapy [8] are the primary anticancer treatments currently being utilized. However more and more studies have shown that these therapies are also associated with numerous postoperative complications, toxicities, and side effects, such as deep vein thrombosis (DVT) [9], upper limb edema [10], myelosuppression [11], liver and renal function, gastrointestinal tract reaction [12], cardiac damage [13], peripheral neurotoxicity, menopause like syndrome [14], or local radiation damage [15]. In addition, breast cancer has an ability to develop resistance to this conventional therapeutics over time [16], and some cancers are insensitive to chemotherapy or radiotherapy [17]. These factors restrict the use of these treatment modalities and impact the prognosis of breast cancer patients. Therefore, it is essential to discover an effective and adjuvant therapeutic agent with low toxicity and fewer adverse side effects for breast cancer treatment.

Traditional Chinese medicine (TCM), an important component of complementary and alternative medicine, evolved almost 3,000 years ago in China with its own unique system of medical theories about pathogenesis, diagnostics, therapeutic principles, and prescriptions [18, 19]. Chinese herbal medicine (CHM) is a mainstay of TCM that mainly consists of medicinal herbs, acupuncture, moxibustion, massage, food therapy, and therapeutic exercise for both treatment and prevention of disease, as well as health protection [20]. CHM has played a positive role in cancer therapy, especially as an adjuvant treatment, which is often used in China to enhance the antitumor effects of Western medicines and protect cancer patients from suffering from adverse treatment effects. These protections include preventing complications due to surgery, reducing toxic effects associated with cancer therapies, alleviating multiple clinical symptoms attributed to cancer, strengthening the body's immunity to prevent recurrence and metastasis, boosting the immune system, prolonging the survival time of postoperation and advanced-stage cancer patients, and improving their quality of life [21–28].

Xihuang pill (XHP), a classic anticancer CHM compound, first mentioned in the ancient Chinese medicine book Wai Ke Quan Sheng Ji, was originally developed by Wang Weide during the Qing Dynasty [29]. XHP is composed of four rare Chinese herbs: musk, bezoar, frankincense, and myrrh. The traditional method of making pills is to mix the four drugs and mash them with steamed yellow rice. Nowadays, capsule formulations of XHP, called Xihuang capsule, has widespread application to meet the increasing clinical demand. In the previous clinical studies, the anticancer activities of both XHP and Xihuang capsule (XHC) have been conducted and reported with positive results for malignancies including breast cancer [30], hepatic carcinoma [31], esophageal cancer [32], gastric cancer [33], colorectal cancer [34], and non-Hodgkin lymphoma [35], as well as bone metastasis [36]. Experiments have demonstrated that this treatment could inhibit cancer proliferation while promoting apoptosis of human tumor cells, prevent tumor invasion and metastasis, enhance immunity, protect against tumor angiogenesis, improve tumor hypercoagulation, and regulate the tumor microenvironment [37–42]. Just because XHP and XHC are equal treatments with equal effects, but just different formulations, they are collectively called XHP in this study.

XHP is a representative example of TCMs which are often used as an adjunct therapy combined with conventional tumor treatment methods such as chemotherapy. As most studies on TCMs are published in Chinese, little is known about them outside of China, thus requiring further research and communication. In the present review, we conducted a meta-analysis to evaluate the efficacy of XHP as a safe adjunctive therapy of chemotherapy for the treatment of breast cancer in comparison with chemotherapy alone, which could provide strong evidence for future clinical decision-making.

## 2. Methods

### 2.1. Database and Search Strategy

We searched for relevant studies published in the following electronic publication databases: Embase, PubMed, Cochrane, Web of Knowledge, the Chinese Biomedical Literature Database (CBM), China National Knowledge Infrastructure (CNKI), Chinese Scientific Journals Database (VIP), China Journal Full-Text Database, and Wanfang Data (for unpublished graduate theses in China) from their inception to August 2018. We executed a comprehensive literature review of randomized controlled trials (RCTs) that combined treatments (Xihuang pill or Xihuang capsule with chemotherapy) for breast cancer patients. The following search terms were used: (Breast Neoplasm OR Neoplasm, Breast OR Breast Tumors OR Breast Tumor OR Tumor, Breast OR Tumors, Breast OR Neoplasms, Breast OR Breast Carcinoma OR Breast Carcinomas OR Carcinoma, Breast OR Carcinomas, Breast OR Mammary Neoplasms, Human OR Human Mammary Neoplasm OR Human Mammary Neoplasms OR Neoplasm, Human Mammary OR Neoplasms, Human Mammary OR Mammary Neoplasm, Human OR Breast Cancer OR Cancer, Breast OR Mammary Cancer OR Cancer, Mammary OR Cancers, Mammary OR Mammary Cancers OR Malignant Neoplasm of Breast OR Breast Malignant Neoplasm OR Breast Malignant Neoplasms OR Malignant Tumor of Breast OR Breast Malignant Tumor OR Breast Malignant Tumors OR Cancer of Breast OR Cancer of the Breast) AND (Xihuang pill OR Xihuang capsule). Studies were restricted to those of human subjects without restriction on language, and the above terms in Chinese were searched in Chinese databases.

### 2.2. Inclusion Criteria

All the studies selected for meta-analysis met the following inclusion criteria: (1) patients in each trials were cytologically or pathologically confirmed as breast cancer; (2) patients received chemotherapy combined with XHP in the treatment group compared to the administration of chemotherapy alone in the control group; (3) RCTs; (4) outcomes included immediate tumor response, quality of life (QoL) using Karnofsky performance score (KPS), immune system response, reduction in adverse reaction of chemotherapy such as myelosuppression, gastrointestinal reaction, and hepatic function damage.

### 2.3. Exclusion Criteria

Studies were excluded due based the following criteria: (1) studies did not meet the above inclusion criteria; (2) use of compounds other than XHP, other traditional Chinese medicine intervention in the treatment group; (3) nonoriginal research or duplicate publication; (4) trials with missing data or documentation of data errors; (5) laboratory studies or review literature.

### 2.4. Data Extraction and Quality Assessment

Two authors (Dan Mao and Lei Feng) independently examined all the titles and abstracts identified as potentially eligible trials, culled obviously unqualified literatures, and then reviewed full texts that might have satisfied the inclusion criteria. Data was extracted from the selected trials into a standard data extract form. The extracted data included first author and year of publication, study size, detail of randomization, age of participants, details of methodology, specifics of the control interventions, durations of treatment, outcome measures, and adverse reactions.

We assessed the methodological quality of each RCT using risk of bias tool in accordance with the Cochrane Hand-book for Systematic Reviews of Interventions. Risk of bias judgment includes six criteria: random sequence generation, allocation concealment, blinding of participants and outcome assessors, incomplete outcome data addressed, free of selective reporting, and other bias based on imbalance of the baseline information. The quality of all included trials was categorized as three potential bias judgments: low, unclear, or high risk of bias. Trials which met all criteria were categorized to low risk of bias, trials which showed that entries met none of the criteria were categorized to high risk of bias, and other trials were categorized to unclear risk of bias if insufficient information was available to make a judgment. All risks for biased data are presented in Figures 2 and 3. Disagreements between the two authors were resolved through consensus or arbitrated by a third author (Siqi Huang).

### 2.5. Statistical Analysis

The articles were managed with EndNote X7, and statistical analyses were carried out using Review Manager 5.3 software from the Cochrane Collaboration. Data were summarized using risk ratio (RR) with 95% confidence intervals (CI) for discontinuous variables or mean difference (MD) with 95% CI calculated for continuous data. Dichotomous data were expressed as relative risk (RR) or odds ratio (OR) with 95% CI. Heterogeneity across trials was tested with the *I*^2^ test. If *I*^2^ ≤ 50% or *P* ≥ 0.1, a fixed model was applied. On the other hand, *I*^2^ > 50% or *P* < 0.1 indicated that a possibility of statistical heterogeneity and so a random-effects model was adopted. The differences between the treatment groups and control groups were considered to be statistically significant when *P* < 0.5.

## 3. Results

### 3.1. Search Results and Study Characteristics

**Figure 1:**
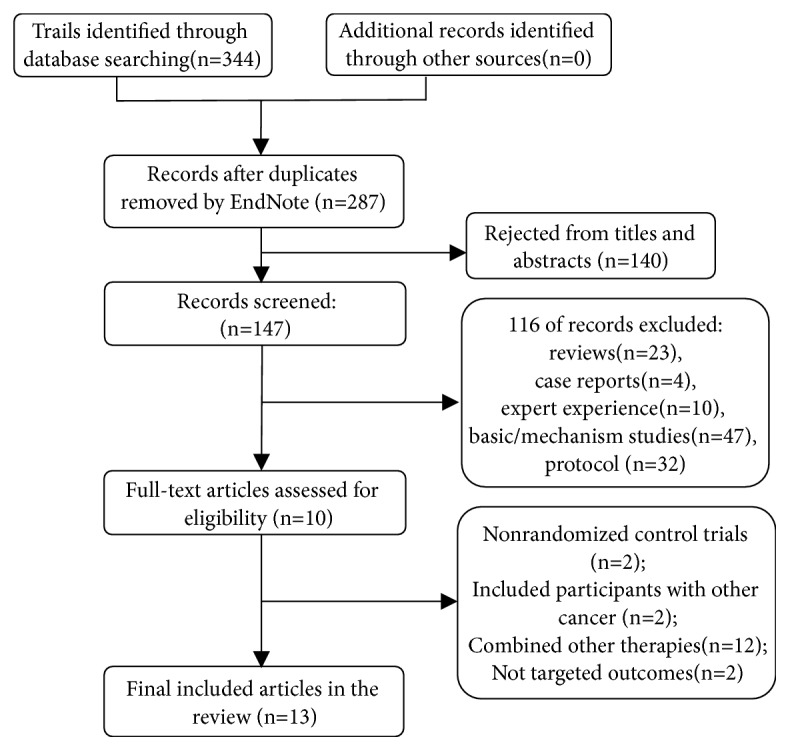
Flow diagram of include studies.

We identified 344 studies through screening of electronic databases. There were 57 studies rejected due to duplication in EndNote X7. After reading titles and abstracts, 147 potentially relevant articles were retrieved. There were 23 literature reviews, 4 case reports, 10 studies were expert experience, 47 were basic/mechanistic studies, and 32 studies were protocols. After further screening, each of these remaining articles was assessed in detail. Eighteen full-text articles did not meet inclusion criteria: 2 studies were not RCTs; 2 studies included participants without only cancer; 12 studies combined other therapies; and 2 studies not investigate targeted outcomes. Finally, a total of 13 studies were included in our analysis (Figure 1) [43–55]. The 13 trials were published between 2010 and 2018 (Table 1). A total of 1272 patients were enrolled in these studies, of which 636 patients participated in chemotherapy combined with XHP and 636 received chemotherapy alone.

### 3.2. Risk of Bias

**Figure 2:**
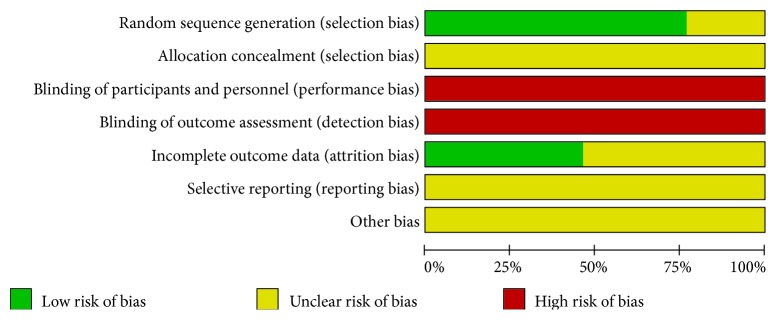
Risk of bias graph.

**Figure 3:**
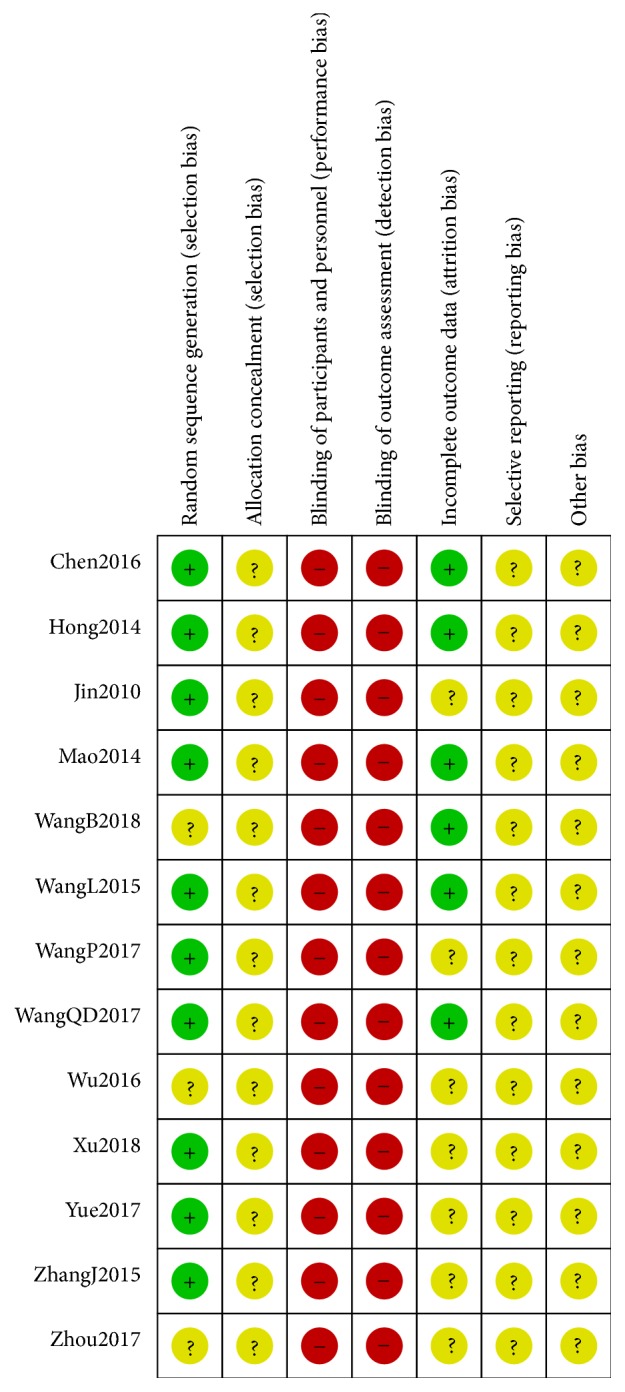
Risk of bias summary.

All patients recruited in the included studies were women with breast cancer, and basically all of the included studies could be evaluated as unclear or high risk in that available data was limited. All trials were described as randomized, with ten trials [43–46, 48–50, 52–54] mentioning a detailed description of the randomization method. Those were considered as low risk as patients were randomly divided into groups. Allocation concealment was not reported in any studies. Attempts to contact the authors by phone or e-mail were unsuccessful. None of the studies gave details about blinding of participants or personnel or blinding of outcome assessment. Six studies described the follow-up process [43, 44, 46–48, 50]; we considered these studies to be low risk. It was not possible to evaluate whether all expected outcomes were reported. And we could not conclude if there were no other biases in each study. Our quality assessment of each methodological parameter is shown in Figures 2 and 3.

### 3.3. Effects of the Intervention

#### 3.3.1. Tumor Response

**Figure 4:**
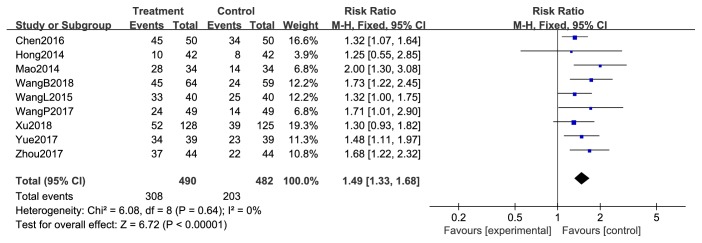
Immediate tumor response during breast cancer treatment (CR+PR).

**Figure 5:**
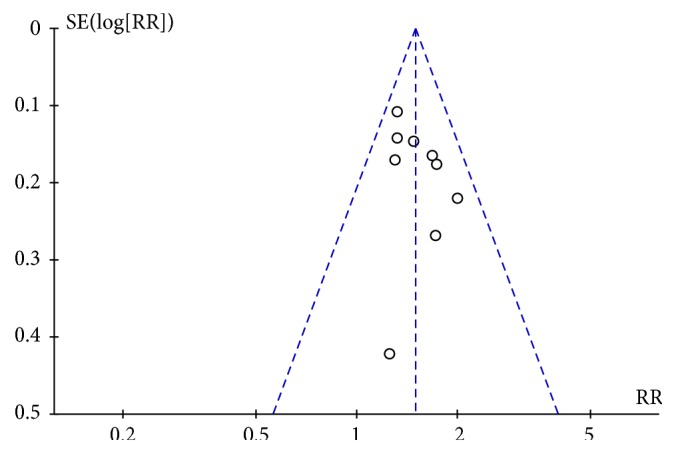
Funnel plot of immediate tumor response during breast cancer treatment (CR+PR).

Results from nine studies stated the tumor response [43–45, 47–49, 52, 53, 55]; 490 patients using chemotherapy combined with XHP were reported to have complete response (CR) or partial response (PR), while 482 patients using chemotherapy only were reported as CR or PR, indicating that the treatment of breast cancer was significantly more effective when chemotherapy was combined with XHP (risk ratio (RR) = 1.49, 95% CI = 1.33-1.68, and *p* < 0.00001, 972 patients). There was no significant heterogeneity among these studies (*χ*^2^ = 6.08; *p* = 0.67; *I*^2^ = 0%) (Figure 4) and a funnel plot was created to indicate publication bias (Figure 5).

#### 3.3.2. Performance Status

**Figure 6:**
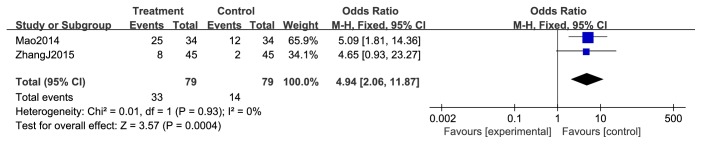
Improvement of KPS during breast cancer treatment.

Changes in Karnofsky performance score (KPS) were analyzed as two types of data in the included studies. The first type reflected the improvement or stabilization of the KPS (ten-point cutoff); the second type was the mean ± SD of KPS data before and after treatment. Only two [46, 54] of the 13 studies, evaluating 158 patients, reported an improvement in KPS. Results from these two studies showed that the combined use of chemotherapy and XHP was significantly related to improved KPS (RR = 4.94; 95% CI = 2.06-11.87; *P* = 0.0004, 158 patients). There was no significant heterogeneity observed among these studies (*χ*^2^ = 0.01; *p* = 0.93; *I*^2^ = 0%) (Figure 6).

**Figure 7:**
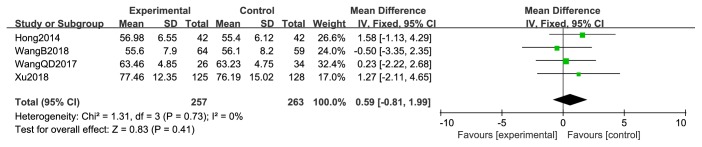
KPS of pretreatment.

**Figure 8:**
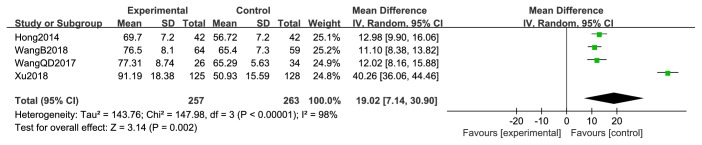
KPS of posttreatment.

Four studies [44, 47, 50, 52] reported pre- and posttreatment KPS. Pretreatment KPS data were not significantly different between the two treatment arms (RR = 0.59; 95% CI: −0.81–1.99; *P* = 0.41; *I*^2^ =0%, 520 patients; Figure 7). However, the pooled results of posttreatment KPS were significantly higher in the XHP combined with chemotherapy group than in the chemotherapy group (RR = 19.02; 95% CI: 7.14–30.90; *P* = 0.002; 520 patients). Heterogeneity among the four studies was low (*χ*^2^ = 147.98; *P* < 0.00001; *I*^2^ = 98%) (Figure 8).

#### 3.3.3. Reduction in Chemotherapeutic Toxicity

**Figure 9:**
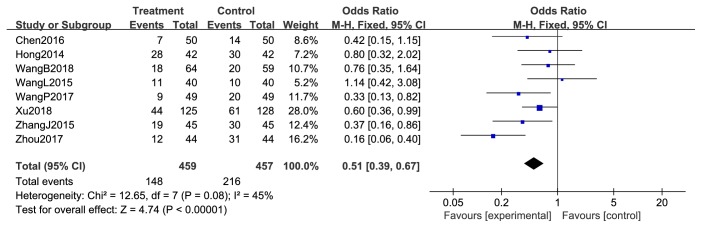
Nausea and vomiting during treatment for breast cancer.

**Figure 10:**
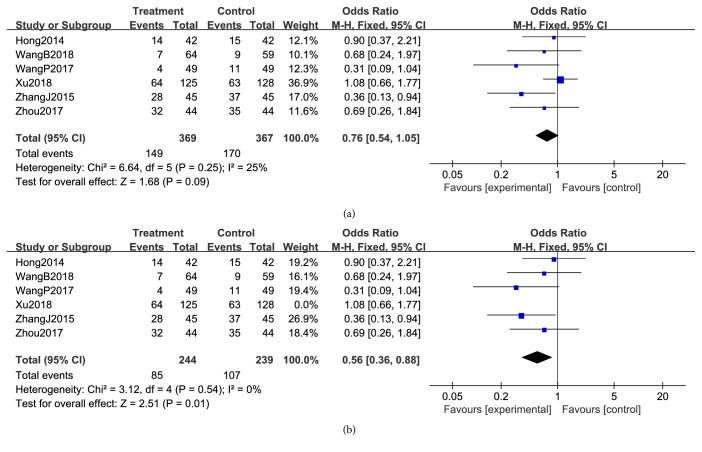
Reductions in WBCs during breast cancer treatment (toxicity grades I-IV).

**Figure 11:**
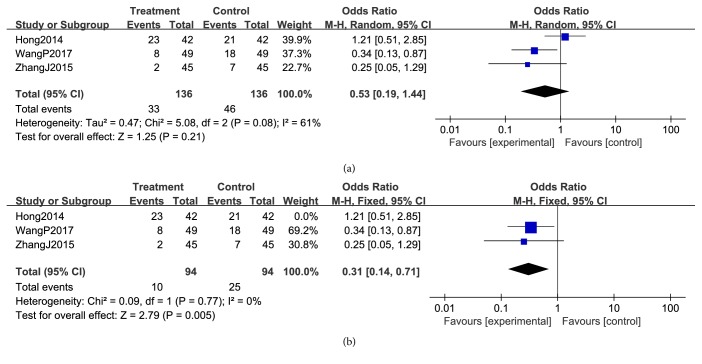
Reduction in platelets during breast cancer treatment (toxicity grades I-IV).

**Figure 12:**
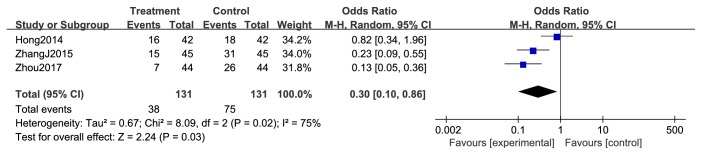
Reduction in hemoglobin during breast cancer treatment (toxicity grades I-IV).

Nausea and vomiting are one of the most distressing adverse events that can occur with chemotherapy [56]. Remarkably, the frequency of nausea and vomiting was reduced significantly in patients treated by XHP combined with chemotherapy (RR = 0.51; 95% CI = 0.39-0.67; *P* = 0.008; eight studies; 916 patients) [43, 44, 47–49, 52, 54, 55]. Heterogeneity testing showed *χ*^2^ = 12.65; *P* < 0.00001; *I*^2^ = 45% (Figure 9). The reduction of grade I-IV WBC inhibition was not significantly different between the two groups (RR = 0.76; 95% CI = 0.54-1.05; *P* = 0.09; six studies; 736 patients) [44, 47, 49, 52, 54, 55] and heterogeneity testing results were *χ*^2^ = 6.64; *P* =0.25; *I*^2^ = 25% (Figure 10(a)). In a sensitivity analysis, by eliminating one study [52], the reduction of WBC inhibition at grades I-IV was significantly less frequent in the XHP combined with chemotherapy group (RR = 0.56; 95% CI = 0.36-0.88; *P* = 0.01; five studies; 483 patients) (Figure 10(b)). The reduction of platelet inhibition at the toxicity grade of I-IV in patients was not significantly different between the two arms (RR = 0.53; 95% CI = 0.19-1.44; *P* = 0.21; three studies; 272 patients) [44, 49, 54]; heterogeneity test results were *χ*^2^ = 5.08; *P* =0.08; *I*^2^ = 61% (Figure 11(a)). In sensitivity analysis, by eliminating one study [44], statistical heterogeneity disappeared (*I*^2^ = 0%). Therefore, fixed-effects model was selected for meta-analysis; the reduction of platelet inhibition at toxicity grades I-IV was significantly less frequent in the XHP combined with chemotherapy group (RR = 0.31; 95% CI = 0.14-0.71; *P* = 0.005; two studies; 188 patients) (Figure 11(b)). Grade I-IV chemotherapy-induced reductions in hemoglobin counts were significantly less frequent in the XHP combined with chemotherapy group (RR = 0.31; 95% CI = 0.19-0.52; *P* < 0.00001; three studies; 262 patients) [44, 54, 55]. The heterogeneity test showed *χ*^2^ = 8.09; *P* =0.02; *I*^2^ = 75% (Figure 12).

**Figure 13:**
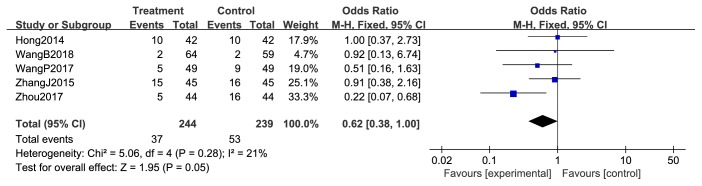
Hepatic function damage during breast cancer treatment.

Five studies [44, 47, 49, 54, 55] reported the chemotherapy-induced adverse reaction of hepatic function damage. And the pooled results exhibited that the frequency of hepatic function damage was not significantly different between the two arms (RR = 0.62; 95% CI = 0.38-1.10; *P* = 0.28; five studies; 483 patients). In the sensitivity analysis comparing the combination treatment with chemotherapy, we saw no treatment benefit with the combination treatment; we detected no significant between-study heterogeneity (*χ*^2^ = 5.06; *P* =0.05; *I*^2^ = 21%) (Figure 13).

#### 3.3.4. Immunoregulation

**Figure 14:**
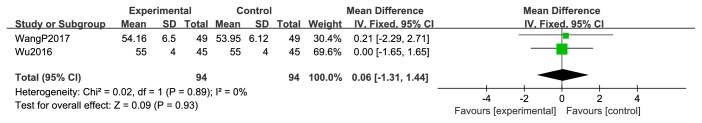
CD3+ of pretreatment.

**Figure 15:**
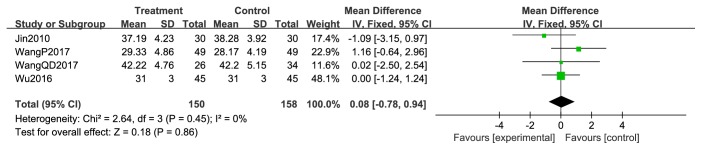
CD4+ of pretreatment.

**Figure 16:**
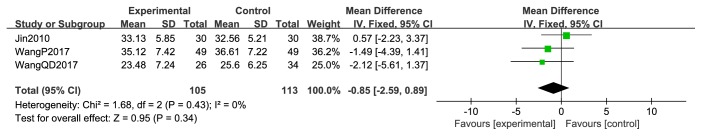
CD8+ of pretreatment.

**Figure 17:**
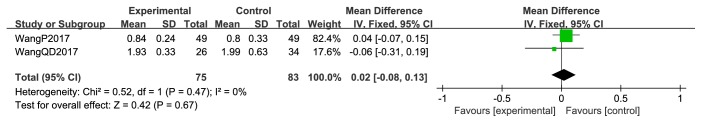
CD4+/CD8+ ratio of pretreatment.

Pretreatment levels with CD3+, CD4+, CD8+, and CD4+/CD8+ cells did not have significant difference between the XHP combined with chemotherapy group and the chemotherapy group (CD3+, RR = 0.06, 95% CI = -0.31-1.44, *P* =0.89, *I*^2^ = 0%; CD4+, RR = 0.08; 95% CI = -0.78-0.94; *P* =0.45; *I*^2^ = 0%; CD8+, RR = -0.85; 95% CI = -2.59-0.89; *P* =0.43; *I*^2^ = 0%; CD4+/CD8+, RR = 0.02; 95% CI = -0.08-0.13; *P* =0.47; *I*^2^ = 0%) (Figures 14–17).

**Figure 18:**
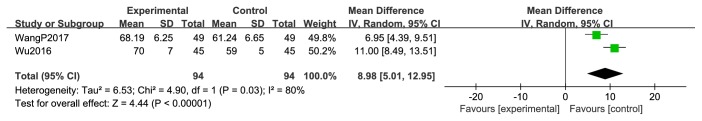
CD3+ of posttreatment.

**Figure 19:**
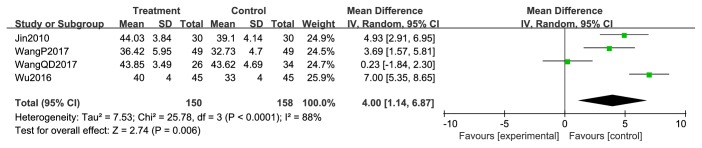
CD4+ of posttreatment.

**Figure 20:**
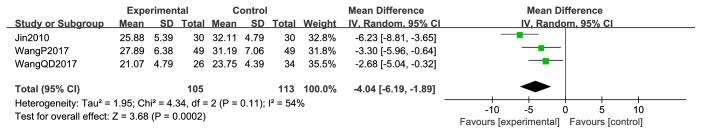
CD8+ of posttreatment.

**Figure 21:**
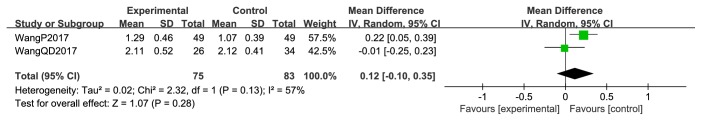
CD4+/CD8+ ratio of posttreatment.

After the treatment of XHP combined with chemotherapy, there was a significant rise in CD3+ cells levels (RR = 8.98; 95% CI = 5.01-12.95; *P* =0.03; two studies; 188 patients) [49, 51]. The heterogeneity testing for this result was *χ*^2^ = 4.90; *P* < 0.00001; *I*^2^ = 80% (Figure 18). Combined therapy also showed a significant advantage in CD4+ cells after treatment (RR = 4.00; 95% CI = 1.14-6.87; *P* =0.006; four studies; 308 patients) [45, 49–51]. Heterogeneity testing showed *χ*^2^ = 25.78; *P* < 0.00001; *I*^2^ = 88% (Figure 19). In addition, there was a significant improvement in CD8+ cell levels in combined therapy group (RR = -4.04; 95% CI = -6.19-1.89; *P* =0.0002; three studies; 218 patients) [45, 49, 50]; heterogeneity testing was *χ*^2^ = 4.34; *P* =0.11; *I*^2^ = 54% (Figure 20). However, posttreatment CD4+/CD8+ levels were not significantly different between the two treatment arms (RR = 0.12; 95% CI = -0.10-0.35; *P* = 0.28; *I*^2^ = 57%; two studies; 158 patients) [49, 50]; heterogeneity testing *χ*^2^ = 2.32; *P* =0.13; *I*^2^ = 57% (Figure 21).

## 4. Discussion

This meta-analysis of 13 RCTs, including 1272 patients, shows that, compared with chemotherapy alone, combination treatment with XHP and chemotherapy had better outcomes, which is evidenced by the significant improvement in the tumor response and performance status among breast cancer patients. Furthermore, combined therapy offers a significant reduction in chemotherapy-induced adverse events, including nausea and vomiting, WBC reduction, platelet reduction, and hemoglobin reduction. These results were strongly encouraging and suggested that the combination of XHP and chemotherapy might be a beneficial clinically therapeutic method superior to chemotherapy alone. These unique advantages could, to some extent, support the use of an integrated TCM and Western approach to medicine in the treatment of breast cancer.

Chemotherapy plays a key role in the systemic treatment of postoperative breast cancer patients, which is a widely used strategy for improving breast cancer survival [57]. Bone marrow suppression, gastrointestinal reactions, hepatic function damage, and immune system destruction are the most obvious chemotherapy-induced side effects [58]. Many patients are unable to tolerate such effects, which can limit its clinical application and impact prognosis. Cancer treatment with chemical agents is destructive to malignant cells and tissues, as well as nontumor tissues. TCM theory holds that the toxicity of chemotherapy may lead to an imbalance of Qi and blood, dysfunction of the viscera, and increased accumulation of pathogenic factors such as toxic heat blood stasis in the body [59].

XHP has many beneficial effects such as heat-clearance and detoxification, activating blood circulation to dissipate blood stasis, and disintegrating scleroma, which was recorded to have effects on treating furunculosis, scrofula, and neoplasms in ancient China [39]. In recent studies, many Chinese medicine experts suggest that XHP could adjust imbalances in the internal body for processes like anti-inflammatory action, reducing temperature, promoting blood circulation, removing toxins, and remarkable antineoplastic properties when complementing chemotherapy against breast cancer [29]. However, most studies on the clinical efficacy of XHP are based on either case reports or expert experience, and it is difficult to reach evidence-based conclusions. This meta-analysis was performed to provide evidence on the usage and justify the clinical application of XHP in breast cancer chemotherapy.

Based on the existing data, we analyzed the mean values of CD3+, CD4+, CD8+, and CD4+/CD8+ ratios in both the XHP combined with chemotherapy group and chemotherapy alone group. Due to mixed quality and the small sample sizes of the included studies, we were unable to clarify whether XHP was part immunoregulation. Although results of these measurements showed that there was a significant enhancement in CD3+ and CD4+ cells levels, as well as obvious suppression of CD8+ cells levels in patients treated with XHP combined with chemotherapy, the change in CD4/CD8 ratio had no statistical significance. Hence, the above evidence is too limited to make a conclusion with confidence. Although the molecular mechanism of action is not fully understood, the improvements in the efficiency of chemotherapy and reductions in chemotherapy-induced adverse events are major advantages for using XHP as an adjunctive therapy in the treatment of breast cancer. The finding that XHP has potential benefits for breast cancer therapy is similar to other reviews [60–63].

There are several strengths and limitations to this study that should be noted. First, we strictly followed the principle of evidence-based medicine to conduct this search, overcame the inconsistency of the included results to provide reliable evidence for the clinical application of XHP. And all reviewers received high-quality training in meta-analyses. One limitation was language bias which was unavoidable because all of the included studies were conducted and published in China. Next, none of the included trials clearly described allocation concealment or blinding processes, which may contribute to high selection risk and performance bias. Third, the lack of multicenter and large size RCTs trials makes it difficult to ignore the low quality of several included studies. Fourth, there was significant heterogeneity in the reduction of WBC inhibition and platelets inhibition; however, sensitivity analysis eliminated the heterogeneity. Differences in sample size, patient age, tumor stage and grade, chemotherapy regimens, and other factors among the studies might also be responsible for the heterogeneity. Additionally, most of the included trials reported positive results. Some negative or nonsensical outcomes selectively unreported may lead to publication bias, which limited integrated analysis. Lastly, only three publications provided information about follow-up. It is therefore impossible to judge long-term efficacy; this flaw may lead to potential biases and influence the final outcomes.

Nevertheless, our findings clearly support the use of XHP in combination with chemotherapy in the clinical management of patients with breast cancer. With the modern extensive application of TCM theories and remarkable therapeutic effects, these methodologies have attracted more public attention and the widespread usage of TCM continues [64]. Accordingly, efforts should be made to conduct more high-level clinical researches such as on medication safety and long-time follow-up to further legitimize TCM worldwide for routine care in the treatment of breast cancer.

## 5. Conclusion

In summary, this meta-analysis demonstrates that XHP could be considered an effective and safe adjunctive treatment to chemotherapy in comparison with chemotherapy alone among breast cancer patients. In addition, XHP was found to have multitarget effects in cancer treatment due to the complex mixture of compound. However, the lack of sufficient molecular evidence still limits the acceptance and application of XHP outside of China. Therefore, further investigation is required to determine the potential mechanisms for antitumor therapeutic effects of XHP. Due to uncertain methodological rules used in many trials, in further studies strict adherence to modern assessment rules will be implemented.

## Figures and Tables

**Table 1:** Characteristics of 13 included trails.

Study	Sample size (T/C)	Control group intervention	Treatment group intervention	Duration (week)	Assessment of outcome
Chen, 2016 [43]	100(50/50)	CAF	XHC+CAF	6	Tumor response, survival time, chemotoxicity

Hong, et al., 2014 [44]	84(42/42)	TAC	XHP+TAC	18	Tumor response, KPS, chemotoxicity, OS, PFS

Jin, et al., 2010 [45]	60(30/30)	CAF	XHP+CAF	6	Symptom curative effect of TCM, CD4+, CD8+

Mao, et al., 2014 [46]	68(34/34)	NR	XHC+Chemotherapy	8	Tumor response, three-year survival rate, KPS, recurrence and metastasis rate

Wang, 2018 [47]	123(64/59)	TP	XHP+TP	12	Tumor response, KPS, tumor marker, chemotoxicity, one-year/two-year survival rate

Wang, 2015 [48]	80(40/40)	CA	XHC+CA	6	Tumor response, MST, PFS, chemotoxicity,

Wang, et al, 2017 [49]	98(49/49)	CAF	XHP+CAF	8	Tumor response, tumor marker, CD3+, CD4+, CD4+/CD8+, coagulation function, chemotoxicity

Wang, 2017 [50]	60(26/34)	AC-T	XHC+AC-T	24	KPS, CD4+, CD8+, CD4+/CD8+

Wu, 2016 [51]	90(45/45)	TEC	XHP+TEC	18	tumor marker, indicators of inflammatory response, CD3+, CD4+

Xu, et al., [52]	253(128/125)	TP	XHP+TP	3	Tumor response, tumor marker, KPS, chemotoxicity

Yue, et al., [53]	78(39/39)	TAC	XHP+TAC	18	Tumor response, P53, HER2, TOPII

Zhang, et al., [54]	90(45/45)	AC*∗*4-T*∗*4	XHC+AC*∗*4-T*∗*4	12	KPS, quality of life, chemotoxicity

Zhou, et al., [55]	88(44/44)	GP	XHC+GP	6	Tumor response, chemotoxicity, TNF-*α*, VEGF, MMP-2, MMP-9

*Notes.* XHC: Xihuang capsule, XHP: Xihuang pill, TG: treatment group, CG: control group, KPS: Karnofsky performance score, OS: overall survival, PFS: progression-free-survival, and MST: median survival time.
